# Poisons and Antidotes

**Published:** 1885-05

**Authors:** 


### Poisons and Antidotes.

Under the head of corrosives, corrosive sublimate stands foremost in importance, being the most typical of this class. The effects are rapid in their development, being well marked by a burning sensation felt in the mouth and throat, followed by agonizing pain in the stomach. The tongue and throat have a white appearance, and excessive tenderness and swelling of the abdomen is noticeable. All authorities agree in recommending albumen in the form of raw eggs —both yolk and white—switched up with a little water, as the best antidote in cases of acute poisoning from corrosive sublimate. The albumen combines with the corrosive sublimate to form an insoluble and comparatively inert compound. Should eggs not be immediately obtainable, gluten obtained from flour, or wheat flour alone mixed with milk or water, may be given until the more reliable antidote is ready. The chief of the corrosive poisons are the mineral acids, sulphuric, nitric and hydrochloric ; the vegetable acids, oxalic, binoxalate of potash, (commonly called salt of lemon and salt of sorrel,) and occasionally in large doses tartaric acid ; the alkalies, potash, soda and ammonia, with certain of their salts, such as pearl ash (commonly called salt of tartar), carbonate of soda (commonly called washing soda), and carbonate of ammonia ; also various metallic compounds, including salts of zinc, tin, silver and antimony, etc. Poisoning by oxalic acid is a very common method chosen by would-be suicides, probably owing to the fact that it is a substance much used in household operations, and therefore readily obtainable by any one bent on committing suicide. In speaking of the action of this poison, that renowned authority, the late Sir Robert Christison, observes in his splendid work on toxicology : "If a person immediately after swallowing a solution of a crystalline salt which tasted purely and strongly acid, is attacked with burning in the throat, then with burning in the stomach, vomiting, particularly the bloody matter, imperceptible pulse, and excessive languor, and dies in half an hour or twenty minutes, or still more in ten or fifteen minutes, I do not know any fallacy which can interfere with the conclusion that oxalic acid was the cause of death.—*Chambers's Journal.*

Tenants may Vacate Unsanitary Houses.—A case has recently been decided in New York justifying the right of a tenant to vacate a house and refuse to pay rent on the ground of unsanitary conditions. The case was : "In a suit for rent claimed to be due from a tenant of a suite of rooms in an apartment house, it appeared that the tenant's wife and servants were taken sick by inhaling a malarial or poisonous gas in the apartsments occupied by them ; that this unhealthy condition of the apartments was owing to a defective condition of the general plumbing work of the house, of which the landlord was notified by orders of the Board of Health, requiring him to have changes made in the plumbing work, and which unhealthy condition could have been removed if he had complied with these orders, that the defendant waited for two weeks, and finding that nothing was done on the part of the landlord, left under the apprehension that he was imperilling the health of himself and family by remaining." The case was appealed to a higher court and confirmed. It is to be hoped the practice will become general.—*Sanitarian.*

